# Water Deficit Stress Alters the Microbial Community Assembly, Structure, and Sources in Corn and Sugar Beet

**DOI:** 10.1111/1462-2920.70186

**Published:** 2025-10-12

**Authors:** Kathryn Bazany, Avinash Dhar, Kristen Otto, Yuan Jing, Pankaj Trivedi

**Affiliations:** ^1^ Microbiome Network and Department of Agricultural Biology Colorado State University Fort Collins Colorado USA; ^2^ Biological and Environmental Systems Sciences Division Oak Ridge National Lab Oak Ridge Tennessee USA; ^3^ Department of Environmental Science and Forestry Connecticut Agricultural Experimental Station New Haven USA; ^4^ Institute of Genomics for Crop Abiotic Stress Tolerance (IGCAST), Department of Plant and Soil Science Texas Tech University Lubbock Texas USA

**Keywords:** microbial community assembly, plant environments, water deficit

## Abstract

Plant‐associated microbes can improve plant fitness under abiotic stress conditions like drought by providing stress‐relieving benefits to the host; however, there is limited research on the complex ways in which microbial communities assemble in plants under varying environmental conditions. In a field study, we examined the bacterial, fungal, and protist communities of the rhizospheres, roots, and leaves of corn and sugar beet grown under irrigated and water deficit conditions. We hypothesised that water deficit would alter the community composition and structure of plant microbiomes by shifting the relative importance of community assembly processes and the patterns of movement from microbial sources to sinks. Using amplicon sequencing and modelling approaches, we found that the water deficit treatment led to key differences in microbial community structure and that these changes were likely driven by differences in community assembly processes and microbial source communities. Altogether, these results indicate that plant microbiome communities are shaped by available microbial sources, host selection factors, microbial interactions, and stochastic forces, and that each of these factors is influenced by osmotic stress. These findings highlight the importance of applying ecological concepts to plant microbiome research in order to elucidate the impacts of environmental factors on microbial community assembly.

## Graphical abstract

Irrigated corn and sugar beet plants and plants grown in water deficit conditions have different microbiome communities across multiple compartments. The path of microbial colonisation of the plant, the dominant ecological processes driving microbiome formation, and multi‐kingdom patterns of co‐occurrence are all influenced by water deficit stress.

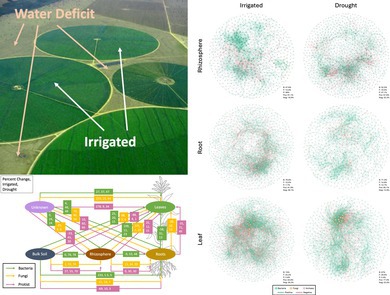

## 1 Introduction

Drought poses significant threats to agricultural systems and food security and is increasing in frequency and severity in many regions due to climate change (Kuwayama et al. 2019; Bahadur et al. 2019; Dai 2013). Drought has caused significant crop losses in the last four decades (Lesk et al. 2016) and is predicted to increase yield losses in the future (Leng and Hall 2019; Pradhan et al. 2022). For example, by the end of the 21st century, drought severity is predicted to increase the yield loss risk of maize, rice, and wheat by 9%–19.4% (Leng and Hall 2019). Drought additionally increases the demand for irrigation, which accounts for 70% of global water consumption. Irrigation demands are projected to increase by 10% by 2050 due to climate change (UN General Assembly, 2nd Committee), meaning that drought‐induced crop loss cannot be mitigated by increased irrigation alone. Additionally, drought poses threats to many natural ecosystems, their biodiversity (Zuo et al. 2022), and the ecosystem services they provide (Deng, Caddell, et al. 2021), so it is important to understand how plants across ecosystems may respond to increased aridity through the progression of anthropogenic climate change (Trivedi et al. 2021). Harnessing the plant microbiome to improve drought resiliency is increasingly considered a viable, sustainable future approach (Trivedi et al. 2022; De Vries et al. 2020; Naylor and Coleman‐Derr 2018). However, optimising the genomic potential of microbiomes as a new platform for enhanced crop production in a drier world requires a detailed understanding of the ecological processes that drive microbiome assembly and dynamics. In order to appropriately leverage the plant microbiome to improve crop resilience to drought stress, we must understand how drought affects the avenues by which microbes colonise the plant host, the dominant community assembly processes, and patterns of multi‐kingdom microbial co‐occurrence in various plant niches.

Plants sculpt the microbial communities within various plant and plant‐surrounding microhabitats, or plant‐associated microbial niche spaces, including the rhizosphere soil (the soil surrounding the plant root and influenced by root exudation), the root environment, and the phyllosphere environment or aboveground plant biomass (Hacquard et al. 2017). The plant‐associated microbiome is mainly derived from soils and gradually enriched and filtered at different plant compartments (Trivedi et al. 2020; Singh et al. 2023); however, other microbial sources like the seed endosphere (Zhang et al. 2022) and the aerobiome, which is especially important in the phylloplane or leaf surface (Xiong, Singh, et al. 2021), can also introduce microbes to the plant‐microbiome system. The relative importance of the factors driving microbiome composition in plant niches varies for different microbial groups (Coleman‐Derr et al. 2016; Hamonts et al. 2018; Xun et al. 2021). For example, plant‐associated bacterial communities are shaped more strongly by the influence of the plant, varying more strongly by niche compartment (bulk, rhizosphere, and rhizoplane), while the fungal community is shaped more strongly by the sampling location and the preexisting soil community (Hamonts et al. 2018; Coleman‐Derr et al. 2016).

Drought can influence the structure and function of the plant‐associated microbiome through various abiotic and biotic changes that include a reduction in water availability, changes in soil chemistry and physical properties, and plant physiology changes. Changes in the soil properties under drought stress can alter the microbiome of bulk soils, the initial pool of microbial recruitment in plant niches (Trivedi et al. 2021; Naylor and Coleman‐Derr 2018). Similarly, changes in the root exudation patterns or immune response to drought can influence the host‐mediated selection responsible for “filtering” effects that allow selective colonisation of different microbial groups in plant microhabitats (Williams and Vries 2020; Singh et al. 2020). While drought‐mediated dysbiosis in the rhizosphere and root bacteria communities is well studied (Naylor and Coleman‐Derr 2018; Santos‐Medellín et al. 2017, 2021; De Vries et al. 2018; Xu et al. 2018, 2021), little is known about the assemblies of different microbial groups along the soil–plant continuum, including root, rhizosphere, and phyllosphere for crops growing in fields under water stress.

Plant‐associated microbial communities form highly intricate ecological networks that constitute numerous interactions between coexisting taxa, belonging to diverse groups in bacteria, fungi, and protists (van der Heijden and Hartmann 2016). Few studies have provided evidence that drought has a significant impact on key properties of microbial networks, including network connectivity and inter‐ and intra‐kingdom interactions (De Vries et al. 2018; Bazany et al. 2022; Vilonen et al. 2023; Lei et al. 2023; Gao et al. 2022). Drought has been shown to have a stronger impact on the bacterial compared to the fungal co‐occurrence networks, suggesting that soil bacterial communities are more vulnerable to drought than fungal communities (De Vries et al. 2018). Generally, drought‐responsive microbial taxa are highly central and connected within networks, suggesting that they are the major drivers of changes in network structure (De Vries et al. 2018; Bazany et al. 2022; Gao et al. 2022). Drought increases the overall positive links between the interacting members, potentially indicating instability in microbial networks (Bazany et al. 2022; Gao et al. 2022). Previous studies have reported the impact of drought on individual microbial networks (De Vries et al. 2018; Peng et al. 2024) or multi‐kingdom interactions in a single compartment (Bazany et al. 2022; Lei et al. 2023), while other studies have examined the impacts on biogeochemical processes like enzyme activity (Sanaullah et al. 2011); there is limited information on the impact of drought on the direction and strength of multi‐kingdom microbial associations for different compartments across contrasting crops.

Plant‐associated microbial communities are shaped by complex ecological processes including selection, dispersal, diversification, and drift (Cordovez et al. 2019). Compared to larger organisms, microbes often possess a greater capacity for dispersal due to their small size, dormancy capabilities which may be selected for in periods of abiotic stress like droughts, and the capability to rapidly evolve due to short lifespans and horizontal gene transfer (Wilkinson et al. 2012; Lennon and Jones 2011; Ochman et al. 2000). While the reproducibility of microbial communities in controlled environments may indicate that plant microbiomes are largely defined by deterministic processes, like homogenous and heterogenous selection (Finkel et al. 2019), this has yet to be extensively verified in the field where more stochastic forces, like drift, dispersal limitation, and homogenising dispersal are in effect (Zhou and Ning 2017). Climate change is postulated to alter the relative importance of ecological processes in controlling microbial community diversity and succession (Trivedi et al. 2022). For example, in grassland ecosystems, experimental warming decreased the relative importance of drift and increased homogeneous selection (Ning et al. 2020; Bei et al. 2023). Little is known about the assembly processes that shape microbial communities in various plant niches under drought stress (Gao et al. 2020; Ning et al. 2020), and how these processes are impacted in different plants and systems. Understanding the impact of drought on community assembly processes would elucidate how microbial communities form under drought. In this study, we aimed to examine how water deficit stress influences the structure, assembly, and co‐occurrence patterns of bacterial, fungal, and protistan communities in the rhizosphere, root, and phyllosphere of the two most important crops of the mid‐Western US, corn and sugar beet. As rainfall in the region is insufficient for the growth of these crops, the unwatered treatment will exert considerable osmotic stress on the plants, which we will be referring to in this paper as water deficit (WD) or “drought”. We hypothesise that: (1) At the community scale, environmental selection of drought‐adapted microbial taxa will alter the community composition of plant‐associated microbial communities; (2) Drought will influence the niche environments and host filtering to alter the paths of colonisation through the plant compartments; (3) Drought will impact microbial community assembly patterns by reducing stochastic processes like dispersal and increasing the prevalence of selection. We believe that dispersal will be limited because many taxa require water for mobility, to move into and throughout the plant, and that selection will increase both due to the direct influence of drought on the microbial communities; (4) Drought‐induced competitive inter‐ and intra‐kingdom microbial interactions will influence the connectedness of the microbial co‐existence network in different plant compartments.

To test our hypotheses, we collected bulk soil, rhizosphere soil, root, and leaf samples from eight paired corn and sugar beet grown under irrigated and water deficit conditions in adjacent fields located across the mid‐western United States (Supporting Information: S1). We used amplicon sequencing to profile the bacteria, fungi, and protists associated with the rhizosphere, roots, and leaves of corn and sugar beet grown under regular irrigated conditions in adjacent fields. We then applied a suite of statistical and ecological modelling approaches to examine the impact of drought on community assembly, source‐sink relationships, and multi‐kingdom interactions in different plant compartments. This research addresses key knowledge gaps in the ways that bacterial, fungal, and protistan communities assemble in the plant rhizosphere, root, and phyllosphere and how these communities and the ecological processes driving their assembly are influenced by drought stress.

## 2 Experimental Procedures

### 2.1 Sample Collection

We collected bulk soil cores and plants (leaves, roots, and rhizosphere soil) from adjacent corn and sugar beet fields in eight sites in Colorado, Montana, Nebraska, and Wyoming (Supporting Information: S1). Two bulk soil cores were collected from each site. Plant samples (leaves, roots, and rhizosphere soil) were collected from both within the reach of the central pivot irrigation boom, called the irrigated treatment, and the planted area beyond the reach of the irrigation machinery, called the water deficit or the “drought” treatment. This commercial watering system was used for the entire growing season from planting to collection. The average yearly rainfall in these sites ranged from 431 to 533 mm, which is insufficient for growing corn or sugar beet without additional irrigation, meaning these plants were grown under dry conditions and experienced significant osmotic stress. The soil in the non‐irrigated treatment had a gravimetric water content of 30%–63% less than the irrigated soils (Supporting Information: S2). This is a similar water reduction to that of greenhouse studies designed to simulate drought (Singh et al. 2021; Puértolas et al. 2017). The irrigated plants were collected between 30 and 40 rows into the field, and the water deficit plants were taken 8 and 12 rows into the field where irrigation did not occur. Three corn and three sugar beet plants were selected from each watering treatment, resulting in three replicates for the plant‐associated compartments (leaves, roots, and rhizosphere soil) and a total of 12 plants per site. Two soil cores were collected outside the zone of planting of each corn and sugar beet field. These cores were subdivided into four technical replicates. All samples were collected in the summer of 2020 during the flowering stage of both species. Samples were placed on ice and shipped to Colorado State University for processing.

### 2.2 Sample Processing

Bulk soil samples were homogenised through a 2 mm sieve and frozen at −20°C until further processing. Plant samples were divided into three niche compartments—rhizosphere soil, classified as any soil tightly bound to the plant roots after roots were thoroughly shaken; root compartment, consisting of the root plane and endosphere (Hamonts et al. 2018); and phyllosphere compartment, consisting of the leaf plane and endosphere. Rhizosphere soil was separated from plant roots using a protocol from Simmons et al. (2018) in which roots were shaken and the remaining soil clinging tightly to the roots was defined as the rhizosphere soil and scraped off. Roots were rinsed with DI water, placed in 50 mL tubes with 40 mL DI water, agitated on a shaker, then rinsed again. Leaves were rinsed to clear soil from the surface, though roots and leaves were not surface sterilised as root and leaf samples are intended as a composite of endosphere microbes and surface microbes (Hamonts et al. 2018). After the soil was removed, roots and leaves were frozen in liquid nitrogen, ground with a mortar and pestle, and kept frozen at −20°C until further processing.

### 2.3 DNA Extraction and Amplicon Sequencing

DNA was extracted from the bulk and rhizosphere soil samples using the DNeasy PowerSoil Pro Kit from QIAGEN (QIAGEN Strasse, Hilden, Germany). From root and leaf samples, DNA was extracted using the DNeasy PowerPlant Pro Kit from QIAGEN (QIAGEN Strasse, Hilden, Germany). DNA quality was assessed with a NanoDrop 2000 (Thermo Fisher Scientific, Waltham, Massachusetts, USA) and DNA quantity was determined with a Qubit Fluorometer (Thermo Fisher Scientific).

Amplicon libraries of the 16S rRNA gene using the 515F/806R primer set (Caporaso et al. 2012), fungal ITS1 region using the ITS1/ITS2 primer set (Caporaso et al. 2012), and eukaryotic 18S regions using the Euk1391f/EukBr primer set (Amaral‐Zettler et al. 2009). Stoek et al. (2010) were prepared using Earth Microbiome Project single barcoded primers and protocols (www.earthmicrobiome.org) to examine the bacterial, fungal, and protist communities, respectively. The DNA was amplified in 30 cycles in 50 μL reactions with Invitrogen Platinum Hotstart PCR Master Mix 2X (Thermo Fisher Scientific). For the amplification of 16S rDNA, we used peptide nucleic acid (PNA) clamps, designed to specifically bind and block the amplification of plastid DNA (pPNA, 5′‐GGCTCAACCCTGGACAG‐3′) and mitochondrial DNA (mPNA, 5′‐GGCAAGTGTTCTTCGGA‐3′) (Lundberg et al. 2013). For 18S, we used PNA clamps designed to block the amplification of the plant host (PNA, 5′‐CATTGGTCGGCTTGTCC‐3′). All samples were pooled in equimolar concentrations and then sequenced on the Illumina MiSeq platform (Illumina Inc., San Diego, California) with a paired‐end protocol at the Next Generation Sequencing Facilities at Colorado State University. More detailed descriptions on DNA amplification are available in Supporting Information.

### 2.4 Bioinformatic Analysis

USEARCH v11 Edgar (2010) and UNOISE3 Edgar (2016) were used to process reads into operational taxon unit (OTU) tables. FastQC was used to determine the quality of each sequencing run (Andrews 2010). Adapters and primers were removed with cutadapt (Martin 2011), and demultiplexed by a single barcode sequence. Once demultiplexed, the sequencing runs were combined with the cat command in Linux. Reads were merged with fastq_mergepairs with a minimum overlap of 200 basepairs (bps) and a maximum mismatch of 20 bps between forward and reverse reads for 16S rRNA, a minimum overlap of 100 and a maximum mismatch of 20 for ITS, and a minimum overlap of 50 and a maximum mismatch of 10 for 18S rRNA. Merged sequence qualities were evaluated and reads with greater than 1 error were removed from the analysis with the fastq_filter command in USEARCH Edgar (2010). The representative datasets were created with UCLUST and UPARSE algorithms (Edgar 2013). Amplicon Sequence Variants (ASVs) were clustered and denoised with DADA2 and with UNOISE3. Sequences with > 97% similarity were clustered into one OTU (Edgar 2016). The De Novo OTU tables were built by aligning the clustered OTUs to the representative datasets with the otutab command in USEARCH. The 16S and 18S ASV tables were assigned taxonomies by aligning sequences to the SILVA database (Pruesse et al. 2007). Protists were defined as all eukaryotic taxa, except fungi and invertebrates (Metazoa) (Delgado‐Baquerizo et al. 2020). Bacterial sequences that match host mitochondria and chloroplast and 18S sequences that matched plant or fungal DNA were removed. The ITS OTU table was aligned to the UNITE database (Nilsson et al. 2019).

### 2.5 Statistical Analysis

Unless otherwise stated, all statistical analysis steps were performed in R (v4.2.1). To assess the influence of water deficit, site, host genotype, and host niche compartment on alpha diversity, the Shannon index of each sample was determined using the vegan package in R (Oksanen et al. 2022). An Analysis of Covariance (ANOVA) test was performed using the Anova() function in the car package on General Linear Models that were built using the glm() function to determine the relative importance of each variable on the Shannon index (Fox et al. 2012). Tukey HSD tests were performed to assess alpha diversity differences by treatment. Shannon diversity was then visualised in boxplots using ggplot2 (Wickham 2011). We ran permutational multivariate analysis of variance (PERMANOVA) using the host compartment, sampling location, and water deficit treatment to establish which one of them is the strongest in shaping the bacterial, fungal, and protistan communities. In cases where irrigation treatment was significant, Canonical Analysis of Principle Coordinates (CAP) analysis was performed using the vegan package and visualised in ggplot2 to determine the influence of water deficit treatment for each microbial kingdom within each plant‐associated habitat. To determine the indicator taxa enriched or depleted under water deficit conditions, we generated volcano plots in DESeq2 (Love et al. 2014).

Phylogenetic trees were generated with MAFFT alignment in QIIME2 with the q2‐phylogeny plugin align_to_tree_mafft_fasttree (Katoh and Standley 2013). OTUs with greater than 20 reads were included in the tree. The .qza files were converted to .nwk with the QIIME2 export plugin. Significantly enriched and depleted taxa were defined as taxa with an FDR < 0.25 determined with MaAsLin2 (Mallick et al. 2021). Trees were visualised in iTOL (Ciccarelli et al. 2006). Source tracker analyses were performed using Fast Expectation Microbial Source Tracking (FEAST) (Shenhav et al. 2019) to determine the proportion of the different potential sources of microbes of each given sink environment. The bulk soil community was used as an overall microbial “source” community. FEAST models were set up under the assumption that the soil microbiome provides the primary source for plant microbiomes. Other microbial sources, like the aerobiome and seed endosphere microbiome, fall under “unknown” sources in our models. We used the iCAMP package (community assembly mechanisms by phylogenetic bin‐based null model analysis), developed by Ning et al. (2020) to determine the relative importance of different community assembly processes in forming the bacterial, fungal, and protistan communities in the rhizosphere, roots, and leaves. As bulk soil samples did not have an associated water deficit treatment, bulk soil was excluded from the iCAMP analysis. Multi‐kingdom microbial co‐existence networks were generated by calculating correlations among bacteria, fungal, and protists OTUs. To minimise the influence of rare taxa, only OTUs with more than 20 reads were kept in the calculation in the data subset. We controlled the false discovery rate by performing 1000 bootstraps on each correlation. The network calculation was performed using SparCC correlations in Python 3 (Friedman and Alm 2012). Different plant compartments have significant differences in both the microbial diversity and community structure. As our aim was to evaluate the impact of drought on the microbial co‐existence networks between two similar compartments, we used different SparCC correlation cutoffs for each plant compartment. A correlation strength of |*r*| > 0.7 was used for the rhizosphere, |*r*| > 0.65 was used for the roots, and |*r*| > 0.5 was used for the leaves with a cutoff of *p* < 0.01 for all niche compartments in order to achieve target network complexity. The networks are being used to explore differences in watering treatment and within niches; the same cutoffs were applied to both treatments. The networks are not intended to be comparable across niches. Networks were visualised in Gephi (Bastian et al. 2009). Topological properties including nodes, edges numbers, degree, and closeness centrality and betweenness centrality were also calculated in Gephi. Further details on the methods used for these statistical tests can be found in the Supporting Information.

## 3 Results and Discussion

### 3.1 Sugar Beet 18S Sequencing Was Dominated by Host Reads in the Root and Leaf Compartments

Overall, paired‐end sequencing resulted in 13,108,603; 8,639,870; 17,649,253 high‐quality reads for bacteria, fungi, and protists, respectively. The reads could be assembled into 33,448; 8445; 10,548 OTUs for bacteria, fungi, and protists, respectively. Samples were singly rarefied to 5000, 5000, and 1000 reads for 16S, ITS, and 18S, respectively, using the MCToolsR package in R (https://github.com/leffj/mctoolsr/). Samples with fewer total reads than the number rarefied to were removed from the analysis. This included 14 leaf samples from 16S, 11 samples from ITS of mixed hosts and sample types, and 112 samples from 18S. We observed a dominance of host reads in the 18S sequencing runs for sugar beet roots and leaves prior to performing the host sequence filtering steps in our bioinformatics pipeline, likely because the PNA used in library prep was designed for monocots and proved less effective in dicots. Filtering out host reads removed the majority of reads from the sugar beet root and leaf samples, resulting in low total reads per sample for these sample types. As a result, corn and sugar beet samples were analysed and reported separately, and we opted to report on the subset in the main figures. Results from the sugar beet analyses are included in the Supporting Information.

### 3.2 Water Deficits Impact the Microbial Community Structure in All the Compartments

**FIGURE 1:**
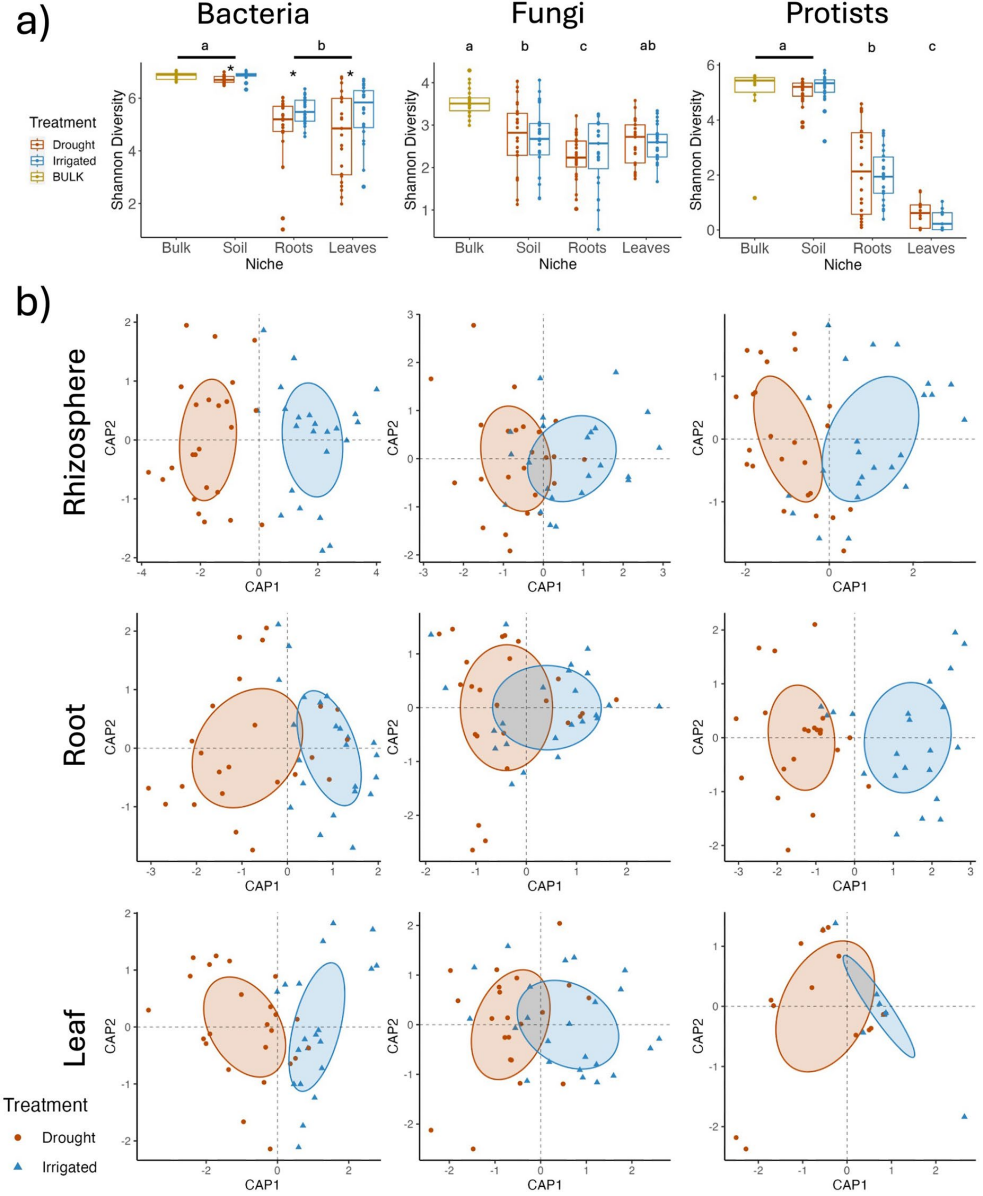
Alpha and beta‐diversity of corn. (a) The alpha diversity (Shannon‐Index) of the different niche compartments—bulk (bulk soil), rhizo (rhizosphere soil), roots, and leaves—and the different irrigation treatments (drought and irrigated) for bacteria, fungi, and protists. Asterix indicated significance (*p* < 0.05) by Tukey HSD test between the irrigation treatments. Letters indicate the significance among the various niche compartments. (b) CAPs ordinations showing the beta‐diversity of the drought and irrigated treatments for bacteria, fungi, and protists (left to right) within the rhizosphere, root, and leaf compartments (top to bottom).

For both plant host species, we observed a significant (*p* < 0.001) decrease in the microbial diversity between soil (bulk soil and rhizosphere) and plant‐associated (root and phyllosphere) environments in corn (Figure 1a; Table 1) and sugar beet (Supporting Information: S3a, S4). Habitat filtering, induced mainly by physical barriers and the plant defence responses, facilitates selective microbial colonisation in the plant compartments, with plants exerting the least selection on the rhizosphere and the most in the phyllosphere (Trivedi et al. 2022; Singh et al. 2023; Xiong, Zhu, et al. 2021). Additionally, soil provides a much wider niche space with a more varied substrate that can host a more diverse community of microorganisms than plant endosphere compartments (Beckers et al. 2017; Dong et al. 2019). While all microbial kingdoms had a significantly reduced alpha‐diversity in the endosphere compartments, protists had a much larger reduction in corn (Figures 1a and S3a), likely because protists are larger organisms and may face greater obstacles in colonising the endosphere compartments. We observed a reduction in the Shannon diversity of bacterial communities of soil, roots, and leaves under water deficit, while no significant changes in any niche compartments for fungi or protists in both hosts (Figure 1a and S3a) were attributed to the watering treatment. Bacterial communities are typically more sensitive to drought than fungi and protists, experiencing reductions in diversity and microbial biomass (Preece et al. 2019; Bazany et al. 2022). Differences in body size, nutrient acquisition potential, cellular metabolism, cell wall structure and constituents, and foraging strategies can lead to the differences in drought tolerance between bacteria, fungi, and protists (Chen et al. 2022; Meisner et al. 2018; Stefan et al. 2014).

**TABLE 1:** Analysis of variance (ANOVA) performed on general linear models (GLMs) of the Shannon Indexes for corn for bacteria, fungi, and protists.

Effect	Bacteria		Fungi		Protist	
	*F*‐statistic	*p*	*F*‐statistic	*p*	*F*‐statistic	*p*
Site	3.806	0.001**	8.995	< 0.001***	3.409	0.003**
Watering	15.737	< 0.001***	0.0398	0.842	0.288	0.593
Niche	62.008	< 0.001***	11.5613	< 0.001***	420.33	< 0.001***
Site:Watering	1.177	0.322	1.128	0.351	4.357	< 0.001***
Site:Niche	1.075	0.389	7.391	< 0.001***	4.482	< 0.001***
Watering:Niche	2.178	0.118	0.674	0.512	1.348	0.266
Site:Watering:Niche	1.296	0.222	1.716	0.063	1.382	0.204

Plant niche, site (growing region), watering treatment, and their interactions explained significant portions of the variation observed in plant‐associated bacterial, fungal, and protistan beta‐diversity for both hosts (Tables 2 and S5). Our results demonstrate that while site characteristics can result in differences in the initial microbial pool, compartment filtering is a dominant determinant of overall microbial community assemblage, in line with previous studies (Deng, Peng, et al. 2021; Xiong, Zhu, et al. 2021; Singh et al. 2023; Durán et al. 2018). Our results further revealed that water deficit treatment significantly influences the community structure of all three microbial groups in each niche compartment and host (*p* < 0.001) (Figure 1b; Supporting Informaton: S3b, S5). In contrast with the significant impact of water deficit on the community composition, non‐significant changes in the alpha‐diversity for fungi and protists in both hosts (Figure 1a; Table 1; Supporting Informaton: S3a, S4) indicate that water deficit drives changes in community composition without altering alpha‐diversity, possibly indicating that water deficit leads to reductions in some taxa while increasing the relative abundance of others. Both bacteria and fungi experience plant compartment‐specific drought‐induced community changes (Santos‐Medellín et al. 2017), though bacterial communities are generally observed to be more influenced by drought than fungal communities (Bouasria et al. 2012). Water deficit induces a range of molecular, physiological, architectural, and developmental responses in plants that can indirectly restructure the microbial community assemblages in plant compartments (Karlowsky et al. 2018; Xu et al. 2021; Williams and Vries 2020). Water deficit can also directly impact microbial communities by selecting for drought‐tolerant species in different plant compartments and in the bulk soil, which serves as a reservoir of organisms from which plants select members of their microbiome (Yandigeri et al. 2012; Naylor and Coleman‐Derr 2018; Ullah et al. 2019).

**TABLE 2:** Permutational multivariate analysis of variance (PERMANOVA) on corn for bacteria, fungi, and protists.

Effect	Bacteria		Fungi		Protist	
	*R* ^2^	*p*	*R* ^2^	*p*	*R* ^2^	*p*
Site	0.15459165	< 0.001***	0.18754221	< 0.001***	0.1558085	< 0.001***
Watering	0.01338723	< 0.001***	0.00928524	< 0.001***	0.01273833	< 0.001***
Niche	0.20706322	< 0.001***	0.30635186	< 0.001***	0.23102539	< 0.001***
Site:Watering	0.04517112	< 0.001***	0.0459203	< 0.001***	0.06334373	< 0.001***
Site:Niche	0.14843698	< 0.001***	0.17614598	< 0.001***	0.22353861	< 0.001***
Watering:Niche	0.0180138	< 0.001***	0.01337471	0.002**	0.01364684	0.002**
Site:Watering:Niche	0.07835945	< 0.001***	0.06462002	< 0.001***	0.05798762	< 0.001***
Residual	0.33497655		0.19675968		0.24191097	

**FIGURE 2:**
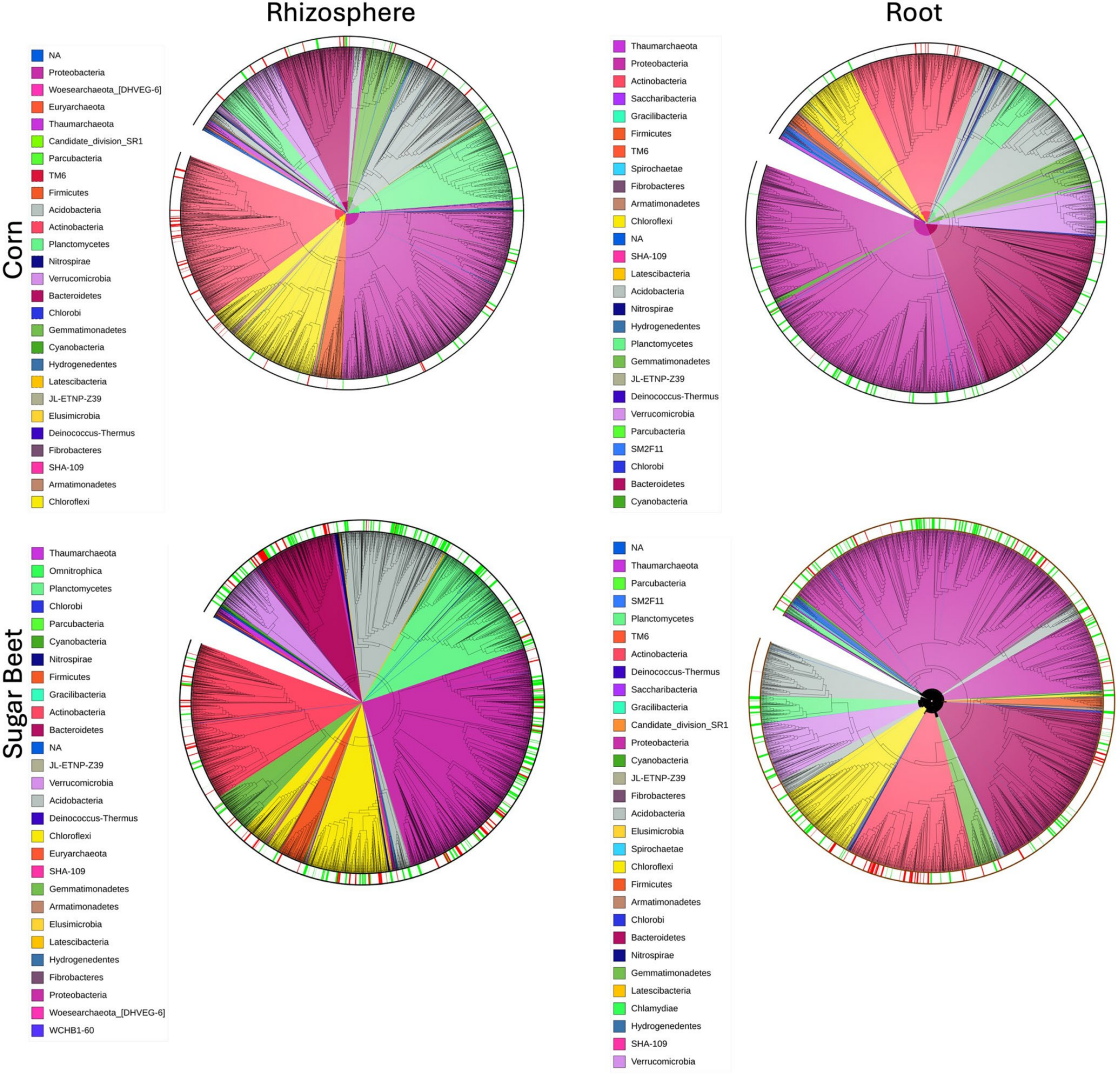
Phylogenetic trees of the bacterial communities in corn (top) and sugar beet (bottom) rhizospheres (left) and roots (right). Trees are colour coded by phylum. Outer ring shows relative enrichment (red) or depletion (green) in drought compared to the irrigated control. Trees were aligned with MAFFT and visualised in iTOL. Significance was determined with MaAsLin2. OTUs with an FDR < 0.25 were considered significantly enriched or depleted with irrigation treatment.

The response of bacterial communities to water deficit is taxonomically consistent across sites. The members of Actinobacteria and Firmicutes were enriched while bacteria belonging to Proteobacteria and Chloroflexi were depleted for water deficit as compared to irrigated treatments in corn (Figure 2). Our results are in line with previous studies that reported the core impact of water deficit on plant‐associated bacterial communities is driven by the enrichment of a few specific lineages (Naylor and Coleman‐Derr 2018; Barnard et al. 2013; De Vries et al. 2020; Yandigeri et al. 2012; Williams and Vries 2020). Monoderms with thick cell walls like Actinobacteria and Chloroflexi are generally drought tolerant or even opportunistic, while more permeable diaderms like Acidobacteria and Proteobacteria experience reductions in drought conditions (Xu et al. 2018; Sun et al. 2020). Many bacteria can form spores to survive extended periods in drought conditions (Filippidou et al. 2016; Schimel 2018). The enrichment of the members of phylum Actinobacteria is not just due to their cellular makeup to tolerate drought conditions, but it is postulated that plants intentionally alter their root exudation profile to actively recruit different Actinobacterial groups—an example of the “cry for help” hypothesis (Rolfe et al. 2019; López‐Ráez et al. 2011; Ait‐El‐Mokhtar et al. 2023; Wang and Song 2022; Xu et al. 2018; Liu et al. 2024). The recruited members of the Actinobacteria phylum can help the plants to tolerate drought stress through the production of osmolytes (Rangseekaew et al. 2022; Niu et al. 2022; Santos‐Medellín et al. 2021), 1‐aminocyclopropane‐1‐carboxylate (ACC) deaminase to reduce stress ethylene, indole‐3‐acetic acid (IAA), an important plant growth hormone (Jog et al. 2014; Zhang et al. 2021; Tamreihao et al. 2016; Chukwuneme et al. 2020; Qin et al. 2017).

For fungi and protists, there were no clear phylum trends with treatment in either host (Supporting Information: S6). In line with earlier studies, our study shows that fungi and protists are generally more drought‐resistant than bacteria (Barnard et al. 2013; Bazany et al. 2022; De Vries et al. 2018). As a whole, fungi and protists are able to persist in drier conditions than bacteria, as they frequently have larger bodies and have mechanisms for desiccation tolerance (Sun et al. 2020; Evans and Wallenstein 2012). Fungi leverage their hyphal networks to obtain water from small soil pores, thereby maintaining nutrient and water uptake over longer distances under water deficit conditions (Guhr et al. 2015; Evans and Wallenstein 2012; Six et al. 2004; Duan et al. 1996; Augé 2004). Because protists generally depend on water for movement through soils, and different types of protists have different drought tolerances, soil protistan taxa are differentially impacted by drought (Stefan et al. 2014). Most soil protists can tolerate the water‐deficit conditions by undergoing a resting stage as cysts (Lennon and Jones 2011; Geisen et al. 2018). While there was a strong overlap in the water‐deficit responsive taxa between different compartments at higher phylogenetic levels, several drought‐responsive taxa were compartment specific. These patterns can arise due to the differences in the bacterial community composition between compartments as well as the differences in the influence of water deficit on the plant‐mediated processes affecting individual compartments. Further research is needed to elucidate taxonomic trends of enrichment for fungi and protists under drought conditions.

### 3.3 Water Deficit Influences the Selection Processes of Plant Microbiome

**FIGURE 3:**
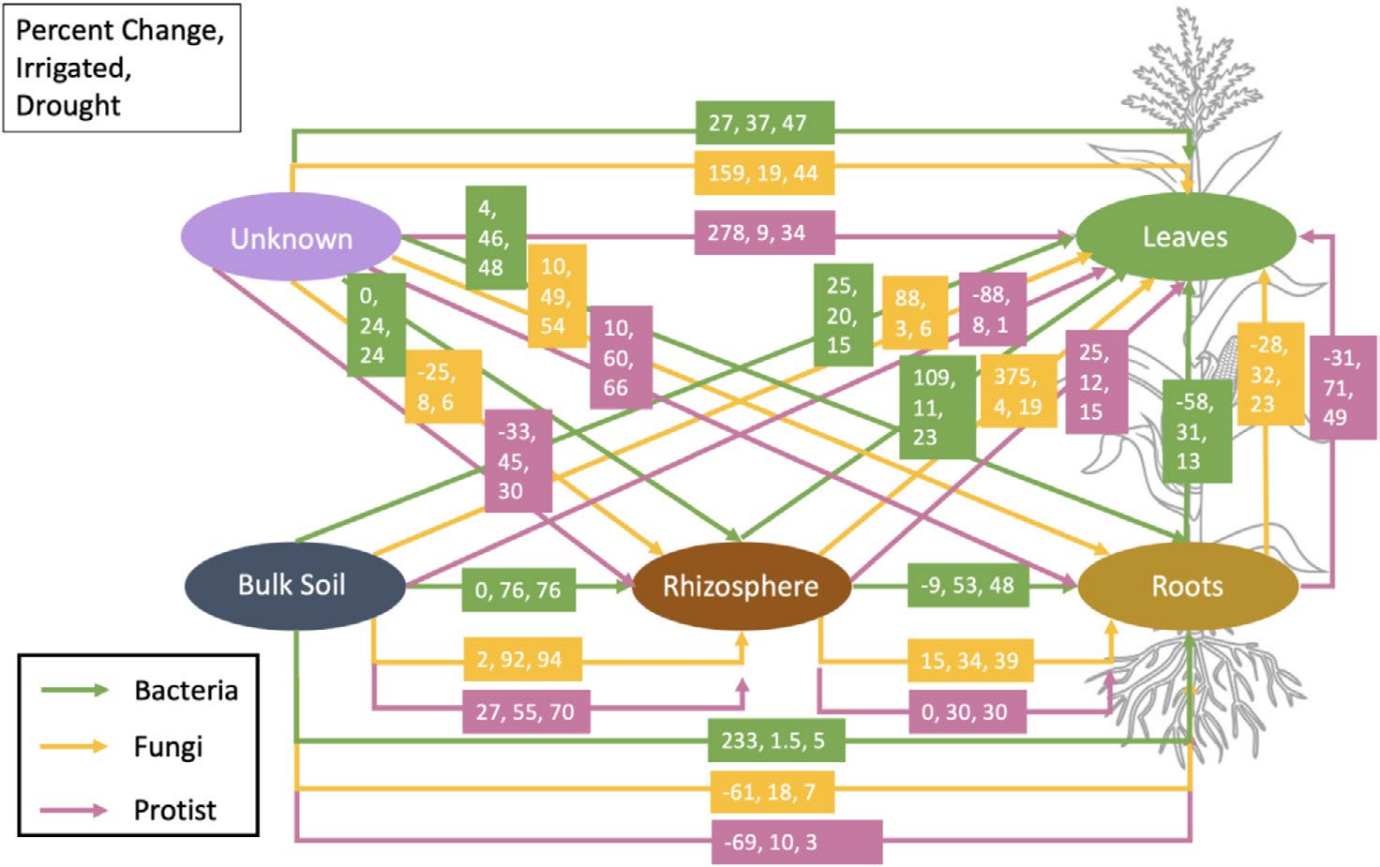
FEAST source tracker analysis showing the path of microbial assembly for bacteria, fungi, and protists in corn. Arrows indicate the proposed direction of microbial colonisation from a source to a sink. The numbers indicate the percent change between treatments [(percent drought—percent irrigated)/percent irrigated], followed by the percent sourced in the irrigated treatment, followed by the percent sourced in the drought treatment.

FEAST based source‐tracking analysis (Shenhav et al. 2019) was conducted to identify the impact of water deficit on the sources of observed bacterial, fungal, and protistan communities in plant environments. For both corn and sugar beet, we observed that plant‐associated microbial communities were mainly derived from bulk soil communities and filtered by host compartment (Figures 3 and S7). The nearest species pool was generally a major contributor to a particular sink. For example, the primary source for the rhizosphere is the bulk soil, the primary source for the root is the rhizosphere, and so on. In general, between 30% and 90% of a given plant‐associated sink was derived from the nearest source, with the known source values greater than 30% in most cases in corn (Figure 2). Our study revealed that water deficit influenced multiple source‐sink relationships of different microbial groups between plant compartments. Within the nearby species source‐sink pools, there were no significant differences in the recruitment of bulk soil microbes to the rhizosphere for bacteria and fungal communities in corn (76% vs. 76% for bacteria and 92% vs. 94% for fungi for irrigated and drought respectively), whereas for protists, there was slightly greater recruitment in the drought treatment (55% vs. 70% for irrigated and drought respectively), though the percentage of root species sourced from the rhizosphere was not affected by watering (30% vs. 30%). There was a higher percentage of root bacteria sourced from the rhizosphere under irrigated conditions (53% vs. 48% for irrigated vs. drought), but the opposite was true for fungi (34% vs. 39% for irrigated vs. drought).

Drought reduced the percentage of leaf microbes sourced from the roots for all three microbial groups in corn (31% vs. 13% for bacteria; 32% vs. 23% for fungi; 71% vs. 49% for protists for irrigated vs. drought, respectively). Drought stress impairs the vascular system of plants, reducing the volume of water in the xylem, the primary transport mechanism of microbes between plant endosphere compartments (Sevanto 2014). Increased contribution of leaf bacteria and fungi from bulk soil and rhizosphere under water deficit indicates direct transfer from the soil sources rather than internal transport from roots. We also observed that the water deficit increased the contribution of unknown sources to the leaf microbial communities, particularly for fungi (19% vs. 44%, irrigated vs. drought) and protists (9% vs. 34%, irrigated vs. drought). The microbial communities on the leaves of fake plants varied with seasonal and environmental conditions, suggesting that the air microbiome is an important source of phylloplane microbes (Xiong, Singh, et al. 2021). Other sources like the native seed endosphere and insects and other pollinators are also likely important sources, as the aerobiome is strongly influenced by the soil microbiome (Zhou et al. 2021). Drought likely increases the capabilities of bacterial and fungal spores to disperse via air due to increased movement of particulate matter (Borlina and Rennó 2017). As many microbes rely on water to move, it stands to reason that the colonisation of the various plant compartments by microbes is impacted by drought. Flagellate and ciliate bacteria and protists rely on water for movement around soil particles and may experience effects in the soil far before aboveground evidence of drought (Stefan et al. 2014). These changes in the relative contributions of microbial sources to various plant‐associated habitats indicate that drought changes the path by which microbes colonise plant hosts and that these paths of colonisation vary by microbial kingdom.

### 3.4 Microbial Community Assembly Processes Are Influenced by Drought

**FIGURE 4:**
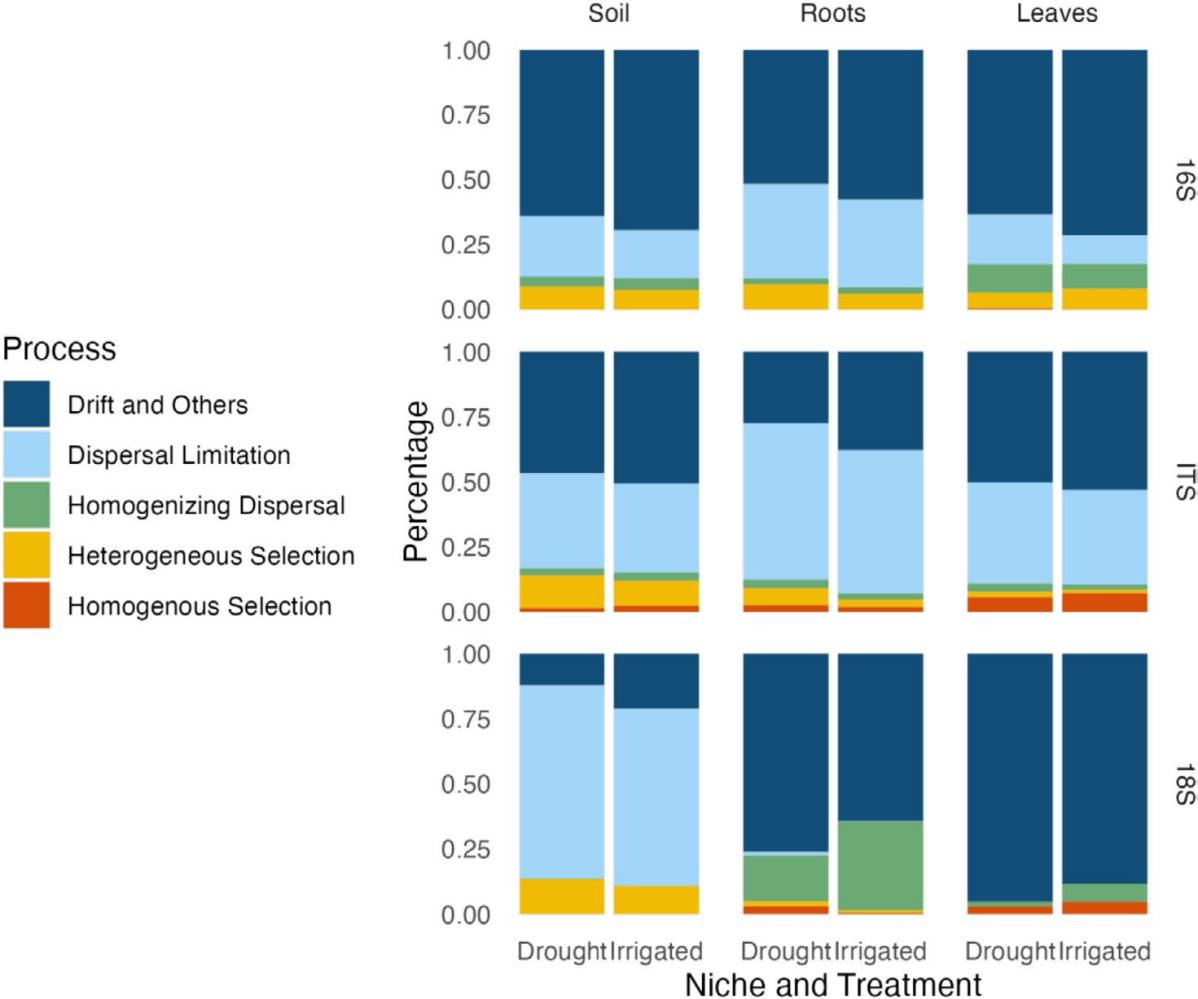
Stacked bar charts representing the results of the iCAMP analysis in corn to determine the prevalence of different microbial community assembly processes in the soil (rhizosphere soil), roots, and leaves (left to right), for the different microbial kingdoms 16S (bacteria), ITS (fungi), and 18S (protists), in the drought and irrigated treatments. The processes tested include stochastic processes (drift and others, dispersal limitation, and homogenising dispersal) and deterministic processes (heterogeneous selection and homogeneous selection).

From the metacommunity perspective, microbial community assembly is a comprehensive result of deterministic and stochastic processes, including selection, dispersal limitation (working in concert with drift), homogenising dispersal, and drift (acting alone) (Leibold et al. 2004; Stegen et al. 2012; Vellend 2010). To determine the impact of water deficit on the ecological drivers controlling the microbial assembly in different plant compartments, we performed iCAMP analysis (Ning et al. 2020). Our results revealed that stochastic processes (drift, dispersal limitation, and homogenising dispersal) were dominant over deterministic processes (homogenous and heterogeneous selection) in controlling the assembly of microbial communities in rhizosphere, roots, and leaf compartments. The overall importance of stochastic processes was surprising given the importance of host selection processes in determining microbial communities of their various niche compartments (Xiong, Zhu, et al. 2021; Wagner et al. 2016). However, the relative importance of host selection varies with the plant compartment (Singh et al. 2023), time (Wagner et al. 2016), and plant growth stage (Xiong, Singh, et al. 2021; de Souza et al. 2016). In general, stochastic processes have a greater influence on early growth stage microbial communities as stochastic factors like arrival order can drive early succession changes (Gao et al. 2020); however, these trends are not universal and may vary for different plant niches and microbial kingdoms. For example, Xiong, Singh, et al. (2021) found that bacterial community assembly of the phylloplane was more deterministic in early growth stages, whereas the influence of deterministic processes on fungi increased over time. As we have collected the plant before the reproductive stage, there is a high likelihood that stochastic processes dominate deterministic assembly processes in our study. The importance of stochastic processes varies with the microbial group and plant compartment (Figure 4; Supporting Information: S8,S9). In the corn rhizosphere, the relative importance of drift and unknown processes was highest for bacteria (69.4% in irrigated corn) followed by fungi (50.7%) and protists (21.1%), while an opposite trend was noticed for dispersal limitation (18.5%, 34.2%, and 68.1% in irrigated corn in bacteria, fungi, and protists respectively). In the roots and leaves, drift was more important than dispersal limitation in controlling bacterial communities (33.8% in roots, 19.2% in leaves of irrigated corn); however, the opposite trend occurred for fungal communities (55.1% in roots, 36.4% in leaves of irrigated corn). Interestingly, the protist communities in the roots and leaves were overwhelmingly controlled by drift and unknown processes (64.3% in roots, 88.3% in leaves of irrigated corn) with a negligible impact from dispersal limitation (0.2% in roots, 0% in leaves of irrigated corn). The differences in the importance of different stochastic processes in controlling the assembly of microbial groups in plant compartments can be linked to differences in the metabolic potential and propagule size within microbial groups. For example, fungi are generally larger and more sedentary organisms than bacteria; therefore, they are more likely to have limited dispersal (Fan et al. 2024). Order of arrival has been shown to have a major impact in shaping fungal microbiomes (Leopold and Busby 2020). Bacteria, due to their small size, fecundity, and comparatively high mobility, are much more likely to be impacted by drift and other stochastic processes than dispersal (Hamonts et al. 2018; Liu et al. 2023; Chen et al. 2023).

Drought is postulated to have differential impacts on community assembly processes. Drift and other stochastic processes decreased in the water deficit treatment for both bacteria and fungi in each niche compartment for corn and sugar beet, suggesting that drought will reduce the random recruitment of fungi and bacteria from the regional species pool (Figure 4; Supporting Information: S8, S9). Selection processes, here heterogeneous and homogenous selection, were higher in the drought treatment for the rhizosphere (7.6% vs. 8.9% for bacteria, 12% vs. 14% for fungi, 10.8% vs. 13.3% for protists in corn in irrigated vs. drought) and root compartments (6.1% vs. 9.7% for bacteria, 4.7% vs. 9% for fungi, 1.4% vs. 4.9% for protists in corn) and lower in the leaves (8.1% vs. 6.6% for bacteria, 8.5% vs. 7.7% for fungi, 4.7% vs. 2.9% for protists in corn) for bacteria, fungi, and protists. This aligns with the source tracking results where drought resulted in a higher prevalence of soil and unknown sources in the leaf niche compared to from the roots which were a more direct source in the irrigated treatments (Figures 3 and S7). The drought‐mediated decrease in the stochastic community assembly patterns in the rhizosphere and roots is likely the result of an increased importance of competitive interactions and host selection in shaping the community composition of rhizosphere soil and root microbiomes under drought stress (Chave 2004; Nemergut et al. 2013). This is likely due to the selective pressure of drought itself on microbial communities, resulting in the elimination or limitation of drought‐sensitive taxa and the proliferation of taxa with a competitive advantage in drought, and to plants selectively recruiting certain taxa under drought stress in their roots and rhizospheres. For example, monoderm bacteria are frequently enriched in the rhizospheres and especially in the roots of drought‐stressed plants (Naylor et al. 2017; Xu et al. 2018). The reduction of selection processes observed in the leaves under drought conditions aligns with the increased percentage of unknown microbial sources found in the source tracker analysis. In drought conditions, soil‐derived taxa are more likely to be directly deposited on the leaf surface, likely mediated by air, allowing for a more stochastically formed community of the leaves during drought (Peñuelas et al. 2012).

### 3.5 Water Deficit Shapes Microbial Co‐Occurrence Networks

**FIGURE 5:**
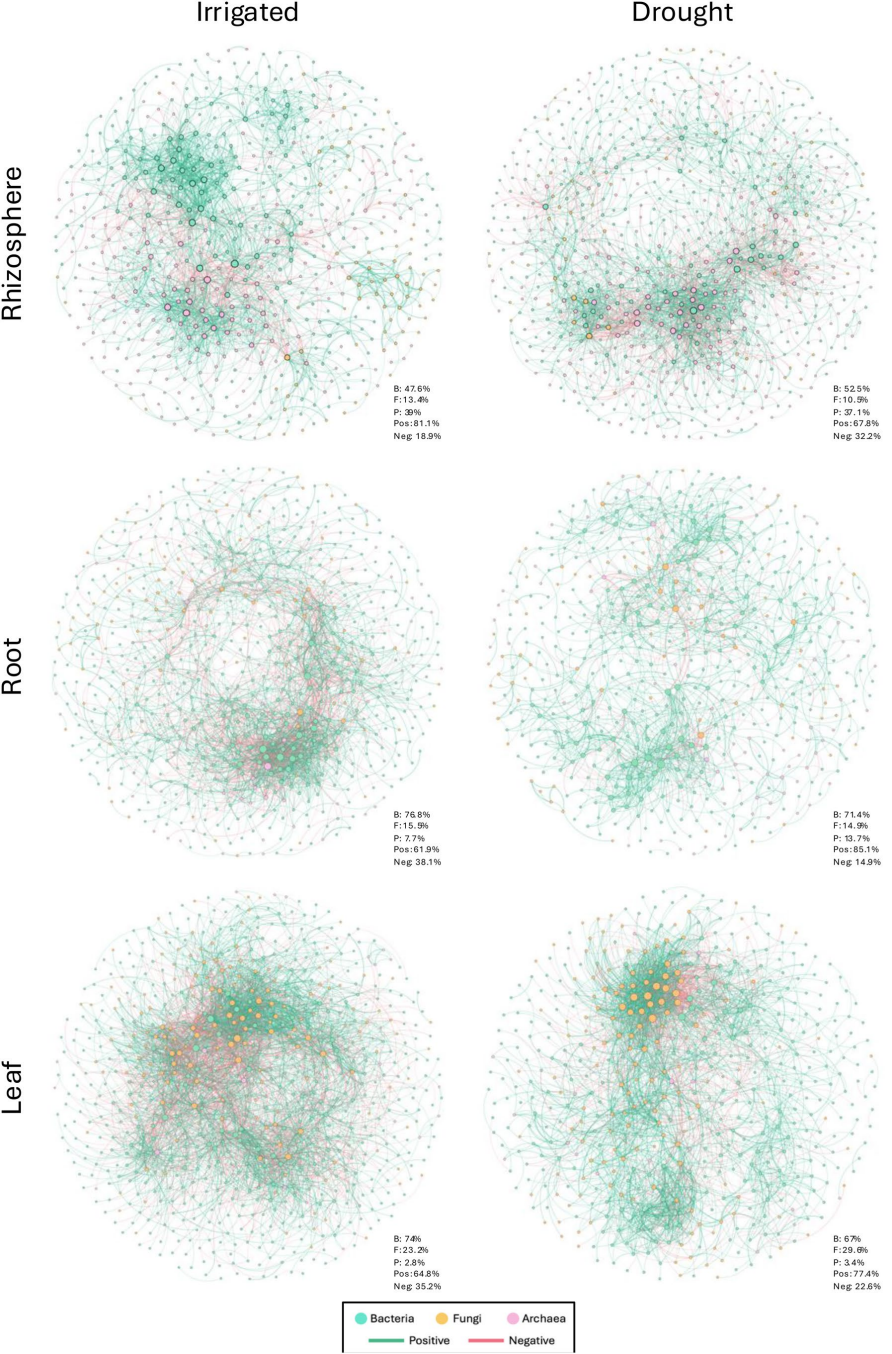
Co‐occurrence networks of the bacterial, fungal, and protistan communities of corn in the irrigated and drought conditions (left to right) for the rhizosphere, root, and leaf compartments (top to bottom). Green points represent bacterial nodes, yellow points represent fungal nodes, and pink points represent fungal nodes. Green lines represent positive correlations between nodes and pink lines represent negative correlations between nodes. The percents at the bottom right of each network show the percent of bacterial (B), fungal (F), and protistan (P) nodes in each network and the percent of positive (pos) and negative (neg) edges. Correlations were performed in SparCC on OTUs with at least 150 reads in the total dataset. Correlation cutoffs were adjusted based on niche compartment to allow for clearer comparison between irrigation treatments. For the rhizosphere networks we kept correlations where |SparCC| > 0.7, for the roots |SparCC| > 0.65, and the leaves |SparCC| > 0.5.

The stability of microbial communities relies on not only the composition, but also on associations that may occur among co‐existing members (De Vries et al. 2018; Gao et al. 2022). Here we used the metacommunity co‐occurrence network based on correlation relationships to examine how drought impacts the co‐occurrence of bacteria, fungi, and protists and plant rhizospheres, roots, and leaves. As expected, the network complexity was highest in the rhizosphere, with root networks being less complex, and leaf networks being far simpler overall for both hosts. In order to emphasise comparisons between the irrigation treatments as opposed to comparisons between niche compartments, different SparCC correlation cutoffs were used for each taxa, where |*r*| > 0.7 was used for the rhizosphere, |*r*| > 0.65 was used for the roots, and |*r*| > 0.5 was used for the leaves (Figures 5, S10, and S11). Interestingly, we found that the response of microbial networks to water deficit differed for different plant compartments, though network complexity and modularity generally decreased with drought. In corn leaves and roots, there was a greater percentage of positive connections in the drought treatment than in the irrigated treatment (77.4% vs. 64.8% in leaves and 85.1% vs. 61.9% in roots). The increases were due largely to correlations between fungi in the leaves and between bacteria in the roots during water deficit, where the relative frequency of positive correlations was increased, and the frequencies of negative correlations were decreased. Increases in the relative frequencies of positive correlations due to drought can weaken the stability of microbial networks (De Vries et al. 2018; Coyte et al. 2015).

In the corn rhizosphere co‐occurrence networks, we observed that the water deficit treatment has a greater percentage of negative connections compared to the irrigated treatment (32.2% and 18.9% in water deficit and irrigated, respectively). Compared to the leaf and root, the co‐occurrence networks of corn rhizospheres had greater contributions from protists in both the drought (3.4%, 13.7%, 37.1% in leaf, root, and rhizosphere, respectively) and in irrigated treatments (2.8%, 7.7%, 39% in leaf, root, and rhizosphere, respectively). In the rhizosphere, the water deficit treatments increased the proportion of connections between protists while decreasing connections between bacteria. For interactions between fungi and other microbial kingdoms in the rhizosphere, there was a greater increase in the number of positive connections in the water deficit (245) than in the irrigated treatment (69). Both bacteria (Barnard et al. 2013; Gao et al. 2022) and protists (Stefan et al. 2014) are reported to be more responsive to water deficit while the fungi are resistant (Guhr et al. 2015; Barnard et al. 2013; Gao et al. 2022). Our results indicate that under water deficit, stronger positive interactions with the resistant groups can allow benefits for sensitive groups towards stress tolerance, though this could also indicate a general reduction in network stability, given the stress gradient hypothesis (Gao et al. 2022). While water deficit increased the overall number of connections between bacteria and protists in the corn rhizosphere (both positive and negative), there was a significant increase in the negative bacteria‐protist connections in the drought treatment compared to the irrigated treatment (577 and 359 in drought and irrigated, respectively). The negative interactions between the bacteria and protists can be the result of competition for similar resources (Hassani et al. 2018) or predation (Gao et al. 2019), as protists can mediate a decrease in plant stress via grazing induced shifts in the rhizosphere microbial communities (Guo et al. 2021; Kuppardt et al. 2018). The impact of environmental stress on microbial co‐occurrence networks is highly variable in both our study and others (Mandakovic et al. 2023). These findings begin to illustrate the complex and varied influences of drought on plant microbiomes, demonstrating that drought can impact co‐occurrence network architecture by mediating changes in multi‐kingdom interactions.

## 4 Conclusions

We examined the impact of drought and host species on the bacterial, fungal, and protistan communities and community assembly processes of corn and sugar beet rhizospheres, roots, and leaves. We found that water deficit stress impacted not only these communities but also the relative importance of different source communities and the ecological processes by which these communities assembled. We recommend that future research consider the influence of host and environmental factors like abiotic stress on the recruitment of certain microbes and how contexts like host species influence these trends. Future research should consider the ecology of microbial communities and their plant hosts as well as the mechanisms behind microbial recruitment to allow for more predictable results on the impacts of employing different management strategies and microbial amendments under varying environmental conditions for different plant species in both agricultural and natural systems.

## Author Contributions

Pankaj Trivedi conceived and supervised the study. Pankaj Trivedi and Kristen Otto designed the experiments and collected samples. Kathryn Bazany led the data analysis with assistance from Avinash Dhar, Yuan Jing, and Pankaj Trivedi. Kathryn Bazany and Pankaj Trivedi wrote the manuscript. All authors read, edited, and approved the final manuscript.

## Conflicts of Interest

The authors declare no conflicts of interest.

## Supporting information


**Data S1:** Supporting Information.

## Data Availability

The data that support the findings of this study are openly available in NCBI SRA at https://www.ncbi.nlm.nih.gov/sra, reference number PRJNA1018526 (SAMN37440405 ‐ SAMN37441335).
