# Supplementary material for: Water Deficit Stress Alters the Microbial Community Assembly, Structure, and Sources in Corn and Sugar Beet
Source: Environ Microbiol. 2025 Oct 12;27(10):e70186. doi: 10.1111/1462-2920.70186 (PMC12515766; doi:10.1111/1462-2920.70186)
Supplement: Supplementary file 1 — Data S1: Supporting Information. [file EMI-27-e70186-s001.docx]

**Supplementary Figures:**

**S1:** Information on the sites including a Site ID, the State, Latitude and Longitude. Each site had a Corn field with a directly adjacent Sugar Beet field watered with central-pivot irrigation.

**
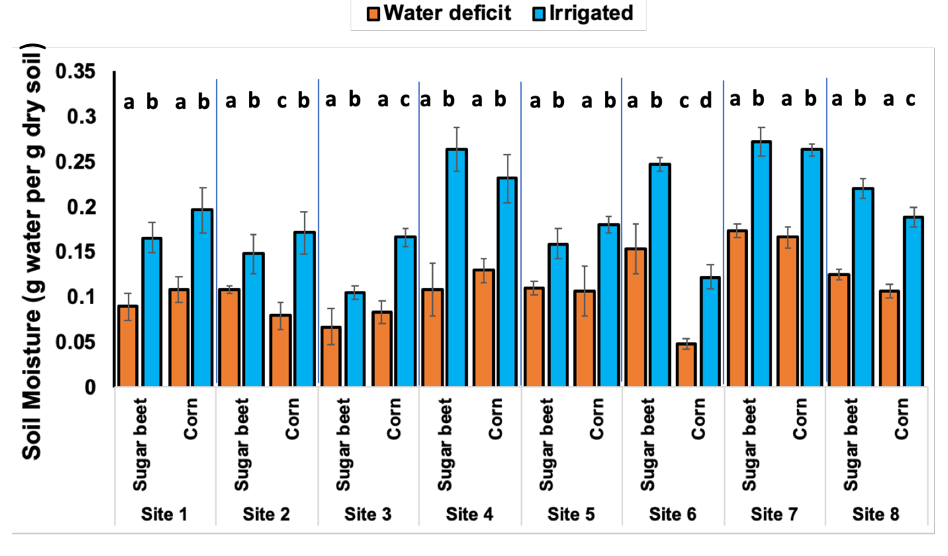
**

**S2:** Differences in soil volumetric water content (VWC, %) between irrigated (blue) and water deficit (brown) treatments for sugar beet and corn at different sites. Different letters mean statistically significant differences at p < 0.05 within each site. Error bars indicate Standard error.


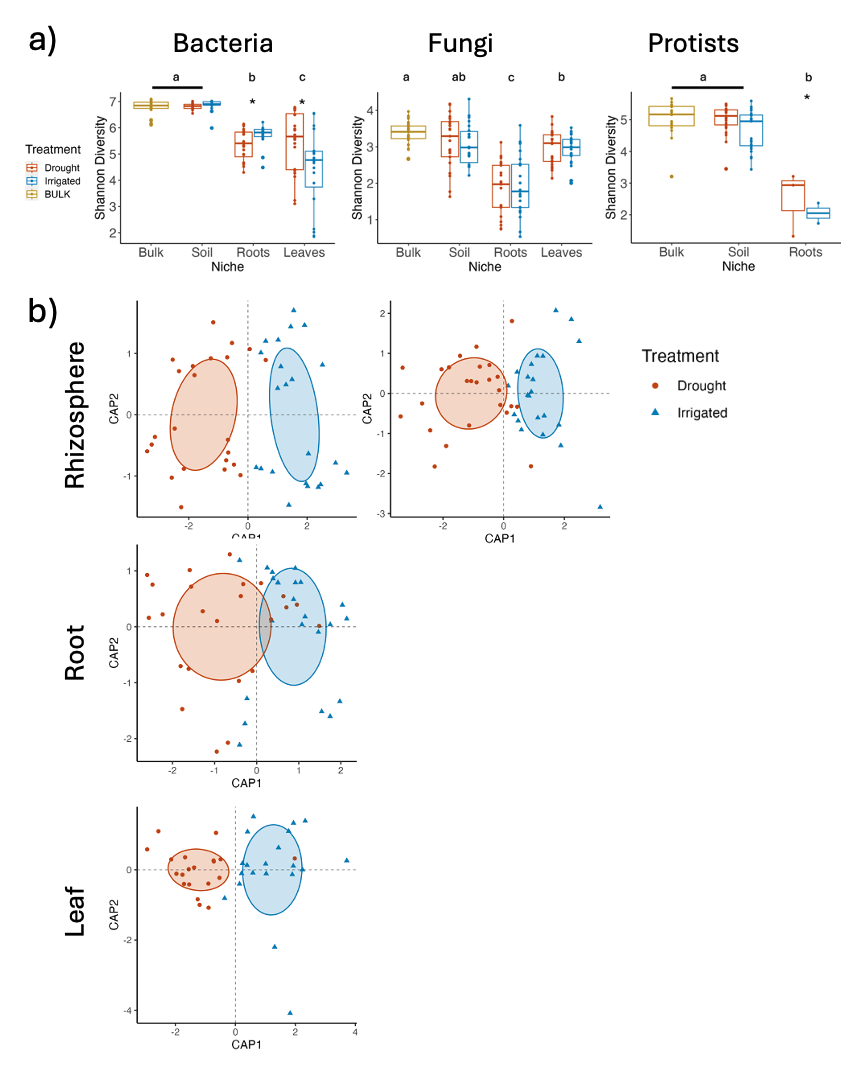


**S3:** Alpha and beta-diversity of Sugar Beet. (a) The alpha diversity (Shannon-Index) of the different niche compartments––bulk (bulk soil), soil (rhizosphere soil), roots, and leaves­––and the different irrigation treatments (drought and irrigated) for bacteria, fungi, and protists. Asterix indicated significance (p < 0.05) by Tukey HSD test between the irrigation treatments. Letters indicate­ the significance among the various niche compartments. (b) CAPs ordinations showing the beta-diversity of the drought and irrigated treatments for bacteria, fungi, and protists (left to right) within the rhizosphere, root, and leaf compartments (top to bottom). Missing CAPs diagrams for the fungal root and leaf and each protist community are due to insufficient data to perform the analysis.

**S4:** Analysis of Variance (ANOVA) performed on general Linear Models (GLMs) of the Shannon Indexes for Sugar Beet for bacteria, fungi, and protists.

**S5:** Permutational Multivariate Analysis of Variance (PERMANOVA) on Sugar Beet for bacteria, fungi, and protists.


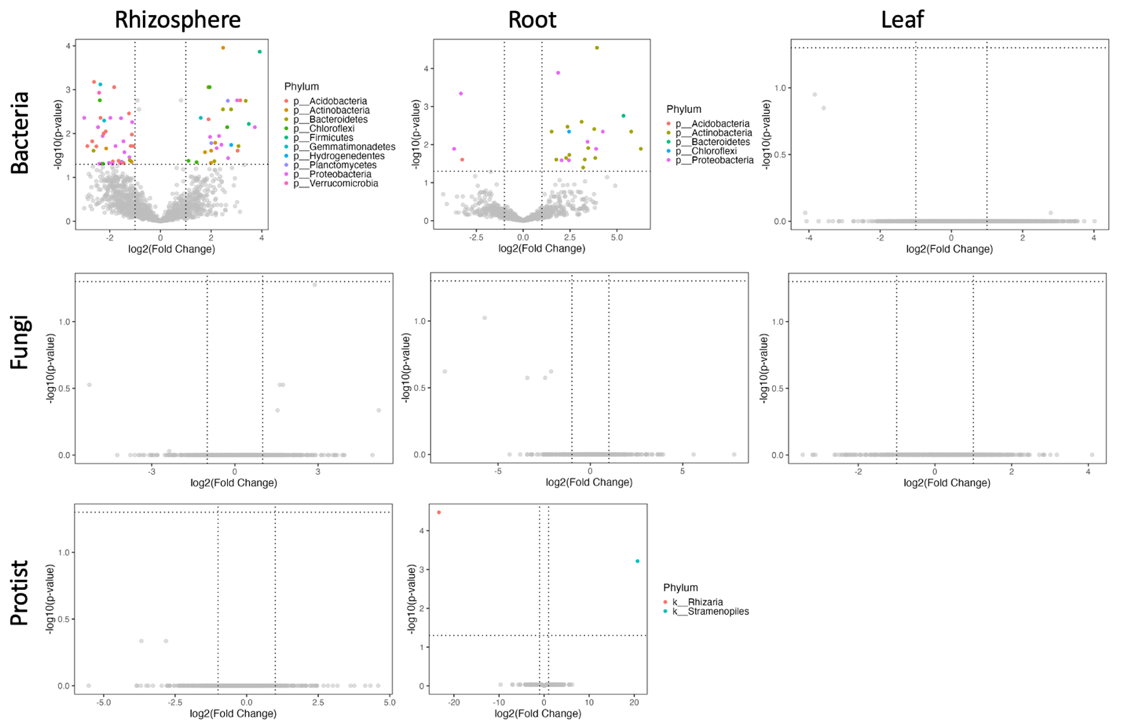

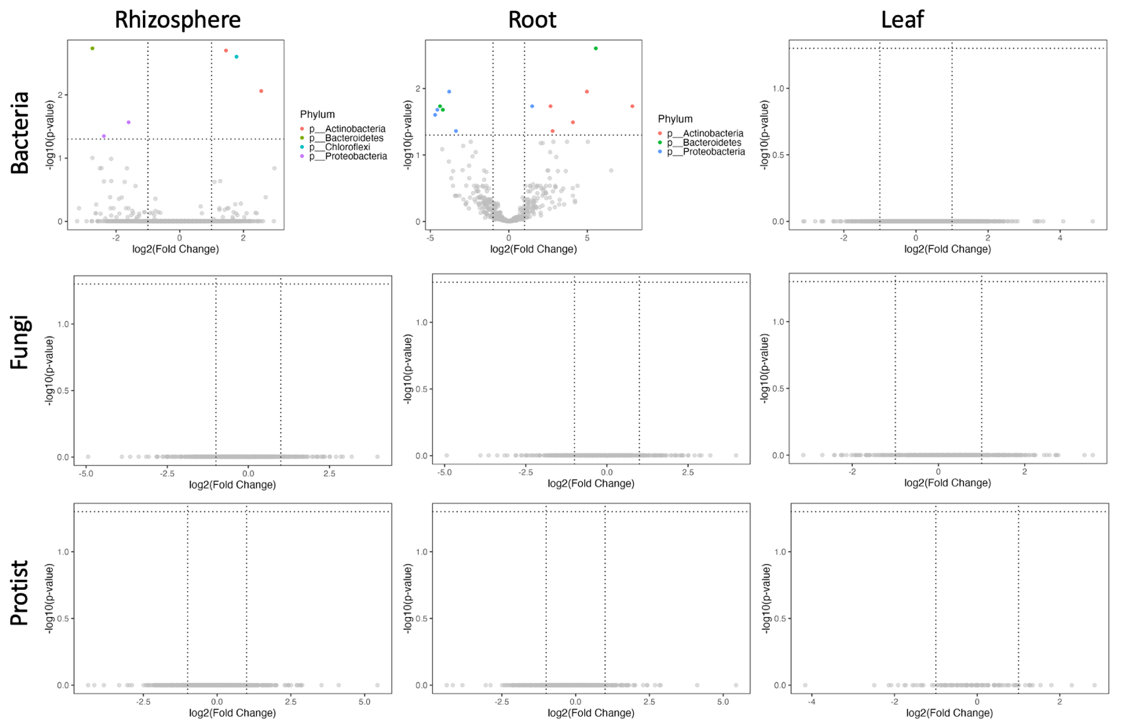
**S6:** Volcano plots for Corn (top nine) and Sugar Beet (bottom nine) within the rhizosphere, roots, and leaves (left to right), for bacteria, fungi, and protists (top to bottom). OTUs with a |log2(Fold Change)| > 1 and a p-value < 0.01 are considered significantly enriched (right) or depleted (left) in the drought treatement compared to the irrigated control and are color coded by phylum.


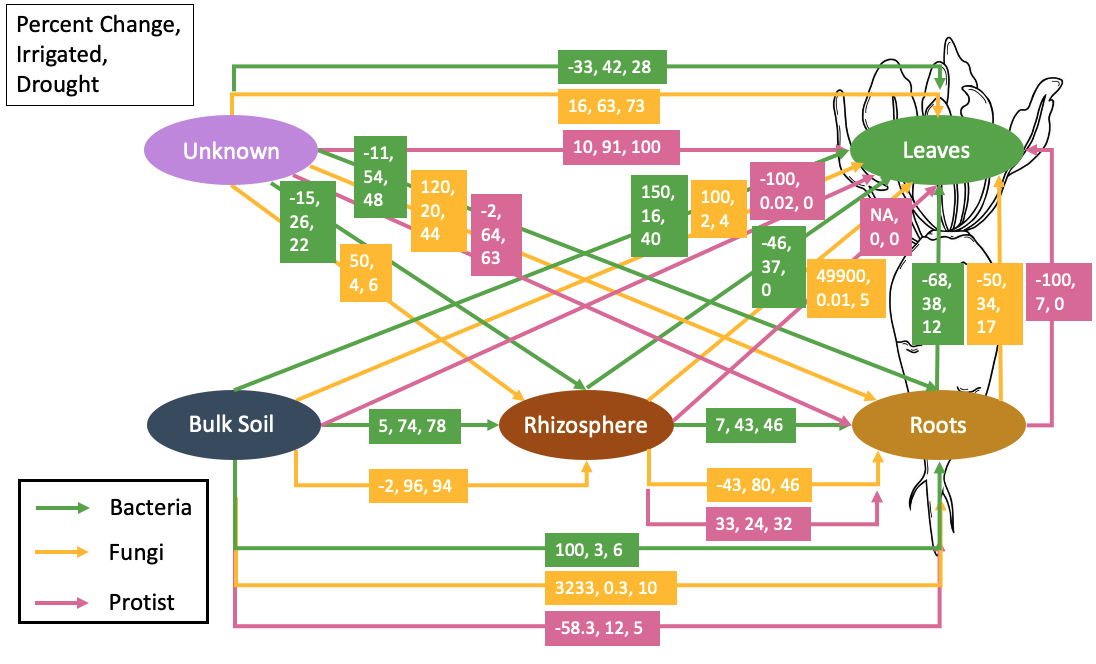


**S7:** FEAST source tracker analysis showing the path of microbial assembly for bacteria, fungi, and protists in Sugar Beet. Arrows indicated the proposed direction of microbial colonization from a source to a sink. The numbers indicate the percent change between treatments [(percent drought – percent irrigated) / percent irrigated], followed by the percent sourced in the irrigated treatment, followed by the percent sourced in the drought treatment.


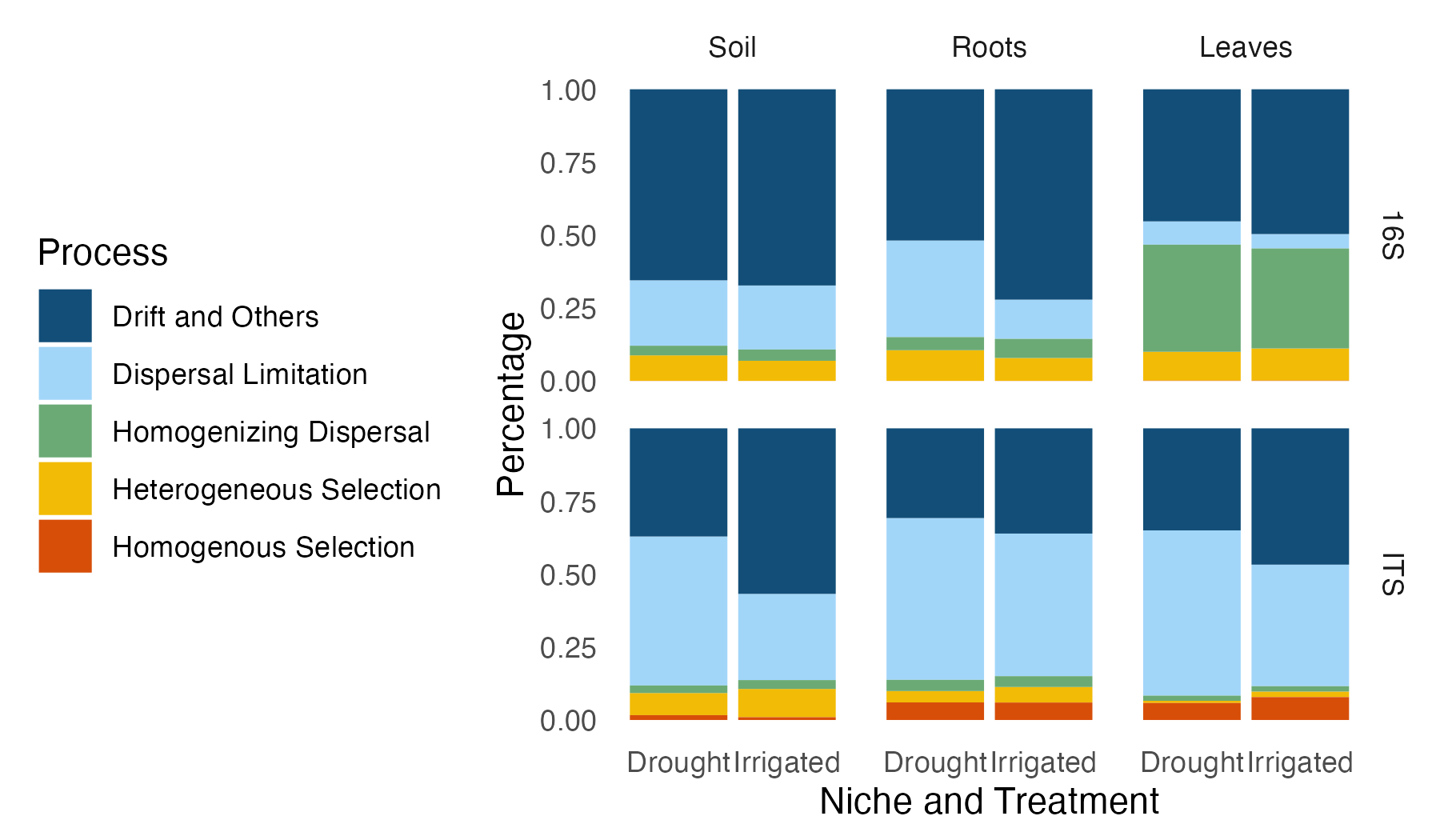


**S8:** Stacked bar charts representing the results of the iCAMP analysis in Sugar Beet to determine the prevalence of different microbial community assembly processes in the soil (rhizosphere soil), roots, and leaves (left to right), for the different microbial kingdoms 16S (bacteria), ITS (fungi), and 18S (protists), in the drought and irrigated treatments. The processes tested include stochastic processes (drift and others, dispersal limitation, and homogenizing dispersal) and deterministic processes (heterogeneous selection and homogenous selection).

**S9:** Percentage (as decimals) of contribution of each community assembly process as determined by iCAMP for Corn (top) and Sugar Beet (bottom) for each amplicon 16S (bacteria), ITS (fungi), 18S (protists) in three niche compartments, leaves, roots, and soil, in the drought and irrigated treatments.


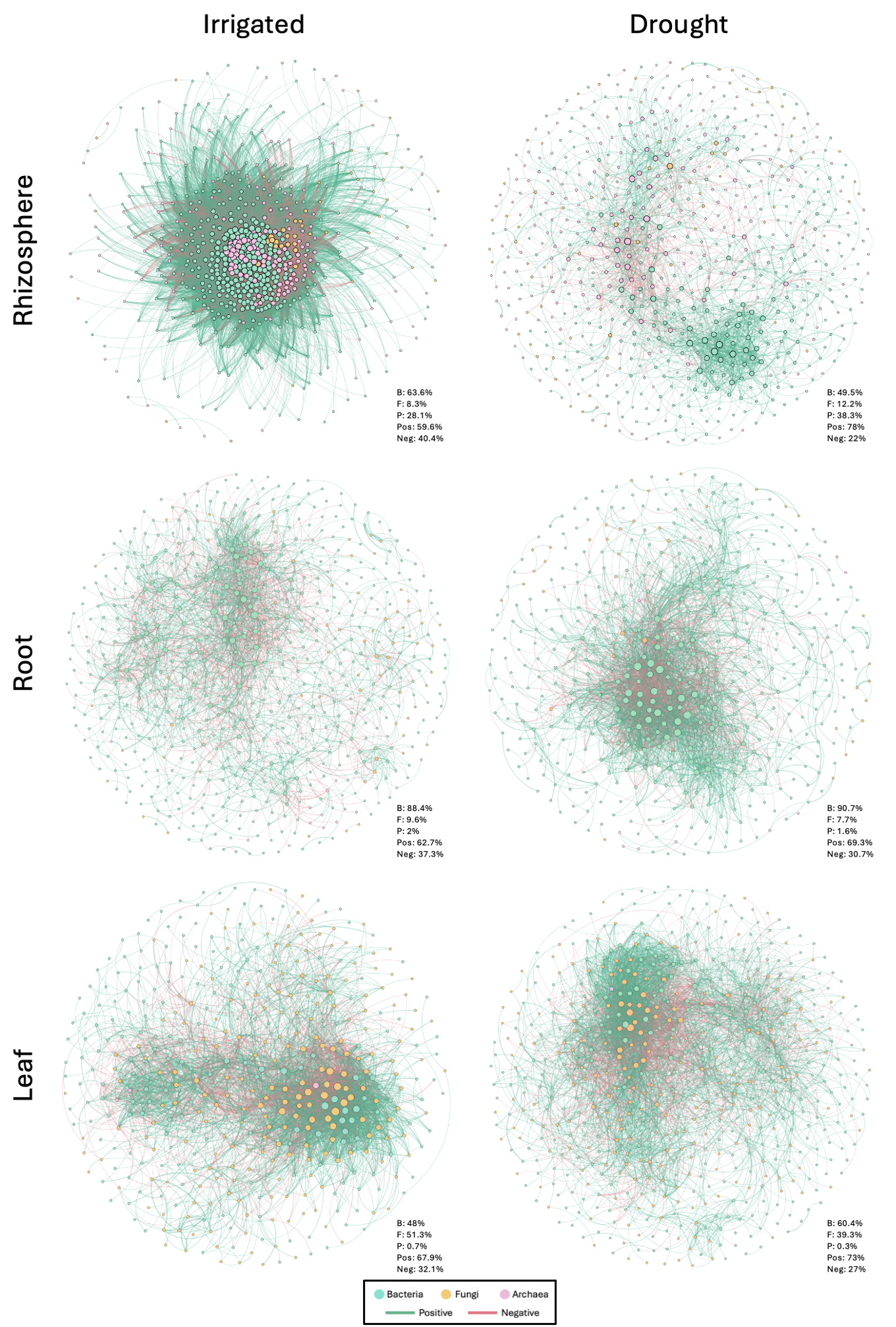


**S10:** Co-occurrence networks of the bacterial, fungal, and protistan communities of Sugar Beet in the irrigated and drought conditions (left to right) for the rhizosphere, root, and leaf compartments (top to bottom). Green points represent bacterial nodes, yellow points represent fungal nodes, and pink points represent fungal nodes. Green lines represent positive correlations between nodes and pink lines represent negative correlations between nodes. The percents at the bottom right of each network show the percent of bacterial (B), fungal (F), and protistan (P) nodes in each network and the percent of positive (pos) and negative (neg) edges. Correlations were performed in SparCC on OTUs with at least 150 reads in the total dataset. Correlation cutoffs were adjusted based on niche compartment to allow for clearer comparison between irrigation treatments. For the rhizosphere networks we kept correlations where |SparCC| > 0.7, for the roots |SparCC| > 0.65, and the leaves |SparCC| > 0.5.


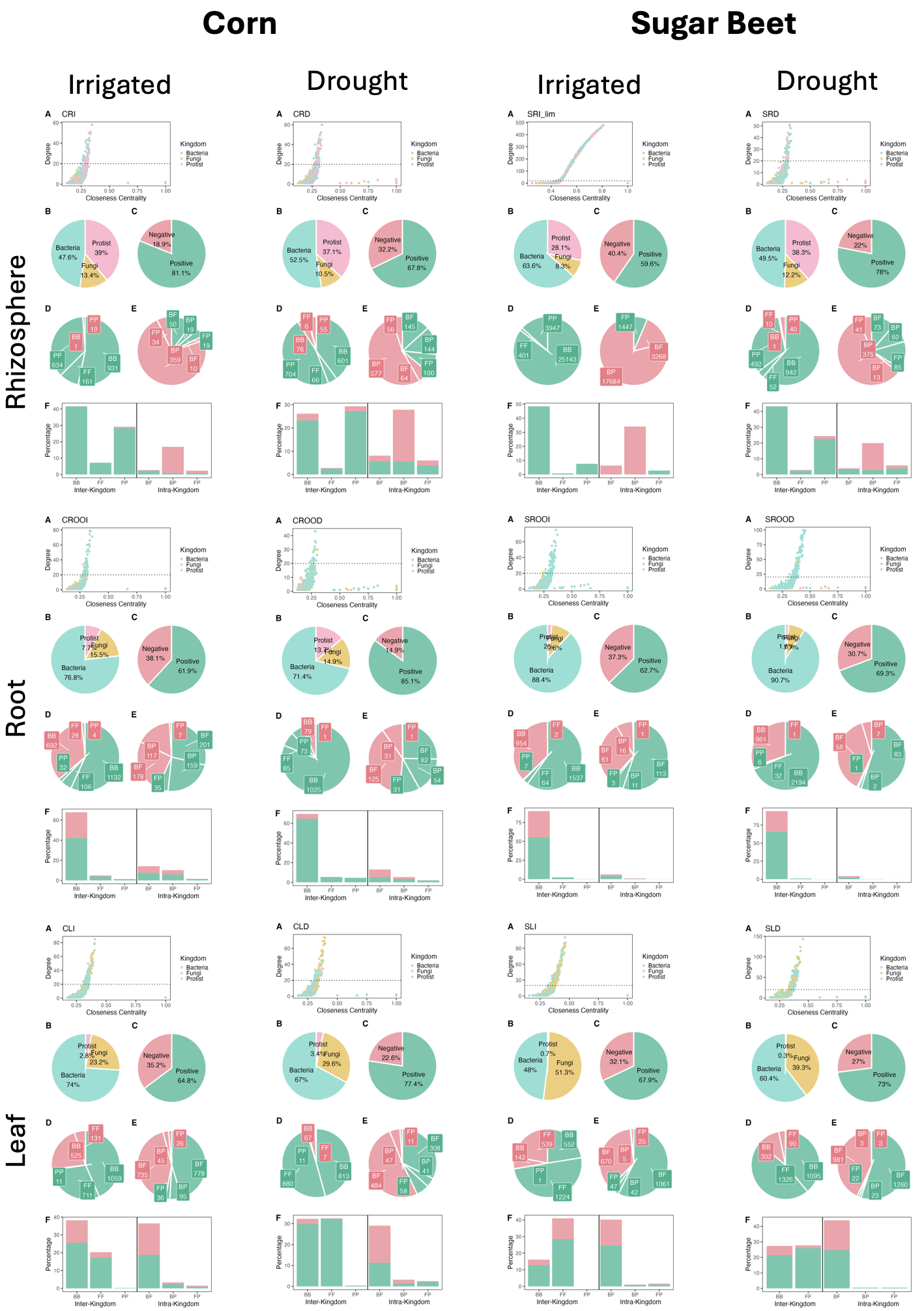


**S11:** Analysis of the co-occurrence networks for Corn (left) and Sugar Beet (right) in the irrigated and drought treatments (left and right) in the rhizosphere, root, and leaf compartments (top to bottom). Within each subset, (a) scatterplot with the degree vs. closeness centrality, where high degree (> 20) and high closeness centrality indicate possible network hubs or keystone species, (b) pie chart with the percentages of each type of node (bacterial, fungal, or protistan) within each network. (c) pie chart with the percentages of positive and negative edges, (d) pie chart with the number of positive (green) and negative (pink) inter-kingdom edges in the network, (e) pie chart with the number of positive (green) and negative (pink) intra-kingdom edges in the network, and (f) bar chart with the percentages of positive (green) and negative (pink) inter-kingdom and intra-kingdom edges between bacterial (B), fungal (F), and protistan (P) OTUs.

**Supplementary Methods:**

Library Preparation:

50 uL PCR reactions were performed for each amplicon. For soil the 16S mix was 25 uL Platinum hotstart 2x, 4 uL of mixed forward and reverse barcoded primers, 4 uL of DNA, and 22 uL of nuclease-free water, and the ITS and 18S mixes were 25 uL Platinum hotstart 2x, 4 uL of mixed forward and reverse barcoded primers, 6 uL of DNA, and 20 uL of nuclease-free water. For the plant tissue the 16S mix was 20 uL of Platinum hotstart 2x, 4 uL of mixed forward and reverse barcoded primers, 4 uL of DNA, 17 uL of nuclease free water, 2.5 uL of pPNA, 2.5 uL of mPNA, both with a concentration of 10 uM. pPNA is a sequence specific peptide nucleic acid for preventing the amplification of chloroplast DNA and mPNA prevents the amplification of mitochondrial DNA. For ITS, the mix was 20 uL of Platinum hotstart 2x, 4 uL of F and R barcoded primers, 6 uL of DNA, 20 uL of nuclease-free water. For 18S, the mix was 20 uL of Platinum hotstart 2x, 4 uL of F and R barcoded primers, 6 uL of DNA, 12.5 uL of water, 7.5 uL of PNA at 10 uM. The PNA sequence was designed to block the amplification of plant host DNA, particularly wheat (Taerum et al. 2020). All amplicons were amplified by heating to 94 degrees Celsius for 2 minutes, followed by 20 replications of 94 degrees for 45 seconds, 54 degrees for 1 minute, 72 degrees for 1 minute 30 seconds, followed by 72 degrees for 5 minutes. All libraries were sequenced on an Illumina MiSeq platform (Illumina Inc., San Diego, California) at the Next Generation Sequencing Facilities at Colorado State University.

Statistical Analysis:

To determine the indicator taxa enriched or depleted under water deficit conditions, we performed Linear Discriminant Analysis Effect Size (LefSe) using the Huttenhower Lab Galaxy portal (Segata et al. 2011). Taxa with a Log10 LDA score < -2 were considered drought indicators, and taxa with a logarithmic LDA score > 2 were considered indicator taxa for the irrigated treatment. The alpha value for the factorial Kruskal-Wallis tests among classes was set at 0.05.

Source tracker analyses were performed using Fast Expectation Microbial Source Tracking (FEAST) (Shenhav et al. 2019) to determine the proportion of the different potential sources of microbes of each given sink environment. Like Source Tracker, FEAST assumes each sink is a convex combination of sources but infers the model parameters via fast expectation-maximization, which is much more scalable than Markov Chain Monte Carlo used by SourceTracker (X. Wang et al. 2023). We aimed to determine the impact of irrigation treatment on the relative importance of each source in determining the microbial community of each sink for bacteria, fungi, and protists. We examined bulk soil as a source for rhizosphere communities, bulk soil and rhizosphere soil as a source for the roots, and bulk soil, rhizosphere soil, and roots as sources for the leaf communities. Unknown sources include microbes from any other source not accounted for in the study including airborne microbes, microbes transferred by insects, and the seed microbiome.

The iCAMP package (community assembly mechanisms by phylogenetic bin-based null model analysis), developed by (Ning et al. 2020), was used to determine the relative importance of community assembly processes. Phylogenetic trees were generated for the 16S, ITS, and 18S reads. Sequences were manually filtered so that only ASVs with at least 300 total reads that were present in at least 20% of samples were included in the phylogenetic tree. This data subset was then aligned with Muscle (R. C. Edgar 2004). Trees were generated with IQ-TREE 2 (Minh et al. 2020). iCAMP analysis was performed on each phylogenetic tree to evaluates the importance of homogenous selection (HoS), heterogeneous selection (HeS), homogenizing dispersal (HD), dispersal limitation (DL), and drift and others (DR) based on beta nearest taxon index (bNTI) and a modified Raup-Crick metric (RC). Deterministic processes were dominant in any given phylogenetic bin where the absolute value of the bNTI was greater than one and stochastic processes were dominant when the absolute value of the bNTI was less than one. From the predominantly deterministic bins, HoS was considered dominant if the bNTI was less than -1.96, and HeS was considered dominant if bNTI was greater than 1.96. In the primarly stochastic bins, HD was considered dominant if the RC was less than -0.95, DL was considered dominant if the RC was greater than 0.95, and DR was considered dominant if the RC was between -0.95 and 0.95. We examined the differences in the relative importance of these processes in determining the bacterial, fungal, and protist communities associated with the different microhabitats of Corn and Sugar Beet under drought stressed and irrigated plants.

Co-occurrence networks were developed based on correlations among and between bacterial, fungal, and protistan OTUs with greater than 20 reads present in the data subset. The SparCC correlations were calculated using Python 3 (Friedman and Alm 2012). The SparCC correlations between every OTU were calculated 100 times. We evaluated the two-sided p-values for each of these 100 datasets with 100 bootstraps. Strong (*r* > 0.70) and robust (*p* < 0.01) correlations were selected and visualized in Gephi (Bastian, Heymann, and Jacomy 2009).
